# PLA2G7, caloric restriction and cardiovascular aging

**DOI:** 10.20517/jca.2022.08

**Published:** 2022-03-16

**Authors:** Fang Cao, Richard T. Lee

**Affiliations:** The Harvard Stem Cell Institute and the Department of Stem Cell and Regenerative Biology, Harvard University, and the Cardiovascular Division, Brigham and Women’s Hospital, Harvard Medical School, Cambridge, MA 02138, USA.

Aging and its related diseases are already major health issues throughout the world, though the mechanisms of aging remain poorly understood. By 2030, the United States population older than 65 years is projected to be over 70 million people, comprising more than a quarter of the total United States population^[1]^. Aging is associated with an increased risk of cardiovascular disease and more severe clinical outcomes, including increased length of hospital stay and mortality^[2]^. Hence there is great interest in molecular pathways of aging, particularly when they are revealed with human relevance.

In remarkable work by Spadaro *et al.*^[3]^ published in the February 10, 2022 issue of *Science*, the authors studied caloric restriction in humans and showed that platelet-activating factor acetylhydrolase (PLA2G7) is an essential mediator of the immune and metabolic changes of aging. Spadaro *et al.*^[3]^ investigated caloric restriction (CR) in clinical trials of healthy middle-age subjects as well as a mouse model of PLA2G7 deletion. They found that PLA2G7 inhibition resulted in reduced age-related thymic dysfunction, adipose tissue inflammation, and liposity. Previously, PLA2G7 has been associated with atherosclerosis, diabetes, and cardiovascular disease^[4]^, and as a marker of cardiac aging^[5]^, thus this study will interest the cardiovascular aging scientific community. Therefore, the work of Spadaro *et al.*^[3]^ suggests PLA2G7 may be a potential target that is a nexus between the immune, metabolic, and cardiovascular pathways of aging.

Spadaro *et al.*^[3]^ studied the effects of caloric restriction on aging by analyzing the thymus and adipose tissue of participants of the Comprehensive Assessment of Long-term Effects of Reducing Intake of Energy (CALERIE)-II clinical trial, in which healthy middle-age subjects voluntarily underwent CR for two years. CR is one of the only known methods to delay aging and predates modern science, having been practiced for over five centuries through various cultures^[6]^. The molecular mechanisms by which CR delays aging may be controlled through key metabolic regulators, such as AMP kinase, mechanistic target of rapamycin, and insulin-like growth factor-1^[7]^. Overall effects of CR include improved metabolic efficiency, reduced adipose tissue fatty deposition, and decreased aging-related inflammation^[8]^. For instance, CALERIE-II participants had reduced adiposity with less inflammation and improved aging-related biomarkers^[8]^. Yet, CR is not without risk. A potential trade-off for delayed aging may be the increased likelihood of infection, postulated to be due to the high metabolic cost of maintaining an active immune system^[9]^. Experimental evidence of this immunocompromising effect is largely seen in animal models with substantial CR (up to 40% reduction in caloric intake)^[10]^. This degree of CR is likely too severe for clinical translation: in the two major clinical trials on caloric restriction (CALERIE and CALERIE-II), healthy volunteers could only achieve 11%−14% reductions in caloric intake even with close supervision and dietary planning^[11]^. It has been unclear whether milder CR would affect immune function in humans as predicted by animal models.

Therefore, to better understand the effects of CR on aging, metabolism, and immune function, Spadaro *et al.*^[3]^ investigated thymic status and abdominal adipose tissue samples of CALERIE-II trial participants. They found that a 14% reduction in caloric intake reduced thymic lipoatrophy and increased CD4/CD8 cell thymus emigration. RNA-sequencing of peripheral blood CD4 cells prior- and post-CR demonstrated similar transcriptional profiles, which supports the idea that mild CR overall maintained or improved immune function. Spadaro *et al.*^[3]^ further investigated CR immune outcomes through the whole-transcriptomic analysis of study participants’ abdominal subcutaneous adipose tissue, with a focus on adipose macrophage populations. Adipose macrophages are well-known contributors to aging through activation of pro-inflammatory pathways^[12]^. Overall, the adipose transcriptomic analysis demonstrated broad changes reminiscent of prior findings in pre-/post-bariatric surgery and twins with discordant exercise habits: reduced markers of age-related inflammatory pathways and increased expression of genes related to fatty acid oxidation and insulin signalling^[3]^. Adipose tissue macrophages were found to have substantially reduced PLA2G7 expression, which is of particular interest given previous research implicating PLA2G7 as an independent cardiovascular disease risk marker.

To elaborate, PLA2G7 is associated with an increased risk of atherosclerosis, cardiac aging, diabetes mellitus, autoimmune disease, and neoplastic conditions - suggesting a role in both metabolic and immune pathways [Figure 1]^[5]^. However, its mechanistic contribution to such a wide array of conditions is largely unclear. PLA2G7 encodes the platelet-activating factor acetyl-hydrolase, which is a component of lipoprotein-associated phospholipase (LP-PLA_2_) and degrades the platelet-activating factor. PLA2G7 expression is upregulated in activated macrophages and atherosclerotic plaque foam cells^[13]^, and LP-PLA_2_ is a component of lipoproteins^[14]^; hence, PLA2G7 is implicated in atherosclerotic plaque formation. However, given that LP-PLA_2_ is found in both high- and low-density lipoproteins, experimental evidence suggests PLA2G7 may have both anti- and pro-atherogenic properties^[4]^. Pharmacological inhibition of LP-PLA_2_ initially suggested a strong anti-atherogenic effect. However, clinical trials with the LP-PLA_2_ inhibitor darapladib failed to show reduced ischemic events in stable coronary heart disease in a Phase III clinical trial (STABILITY TRIAL)^[15]^. Overall, PLA2G7 has been extensively explored in coronary and cardiovascular disease due to its upregulation in activated macrophages and its potential role in cardiovascular aging.

Given that the decrease in PLA2G7 occurred in adipose macrophages, Spadaro *et al.*^[3]^ hypothesized that PLA2G7 played a role in both metabolic and immunogenic disease pathways. Therefore, they generated a genetic deletion mouse model of *PLA2G7* (*PLA2G7*^*−/−*^) and found that PLA2G7^−/−^ protected mice from high-fat diets, leading to reduced weight gain and less relative fat mass^[3]^. These changes may be attributed to increased metabolism, for *PLA2G7*^*−/−*^ mice were found to have increased total daily energy expenditure with higher rates of adipose tissue lipolysis. Indeed, aged *PLA2G7*^*−/−*^ mice were found to have increased expression of genes involved in fatty acid oxidation, recapitulating the transcriptomic findings from the clinical trial analysis^[3]^. Additionally, *PLA2G7*^*−/−*^ mice had reduced pro-inflammatory markers with fewer pro-inflammatory leukocytes; lower macrophage expression of interleukin-2 (IL-2), interleukin-6 (IL-6), tumor necrosis factor-alpha (TNF-α); and reduced ceramide activation of NLRP3 inflammasome. In contrast, other inflammasome activation pathways (NLRC4, AIM2) were largely unaffected. The fact that PLA2G7 deletion specifically stunted ceramide activation of the NLRP3 inflammasome is supported by existing literature^[16]^ and is significant because this pathway is implicated in obesity-related inflammation. Ceramide is an obesity-related molecule, and its activation of NLRP3 induces inflammation with significant downstream consequences, including increased hepatic steatosis and cardiac energy expenditure^[17]^. Spadaro *et al.*^[3]^ then demonstrated that aged *PLA2G7*^*−/−*^ mice, compared to control, had significantly reduced thymic lipoatrophy and higher thymocyte count, recapitulating findings in the CALERIE-II clinical trial. Ultimately, the authors demonstrated that PLA2G7 ablation specifically reduced age- and metabolism-induced inflammation while preserving and potentially improving immune function and organ deterioration in old age.

The role of PLA2G7 in the context of cardiovascular aging has not yet been elucidated. PLA2G7 has been extensively studied in the context of atherosclerotic cardiovascular disease and has been postulated as a marker in cardiac aging^[5]^. Cardiac aging involves reductions in resident anti-inflammatory macrophages, and an increase in blood-monocyte-derived pro-inflammatory macrophage populations that may induce increased oxidative stress and reduced production of nitric oxide, amongst other detrimental effects^[18]^. The role of PLA2G7 on cardiac immunosenescence and its downstream consequences for cardiac aging is currently unknown. The results of this study provide grounds to investigate the effects of PLA2G7 and caloric restriction on cardiac aging. This can be performed with aged *PLA2G7*^*−/−*^ mice and commercially available inhibitors of PLA2G7 (e.g., darapladib). While darapladib did not clinically reduce the risk of coronary heart disease^[15]^, this study adds to further research completed recently on PLA2G7. For instance, *post hoc* analysis of the STABILITY trial suggested that a subgroup of diabetics may benefit from PLA2G7 inhibition, especially if they are known to have genetic variants that increase Lp-PLA_2_ activity^[19]^. Similarly, PLA2G7 may also play a potential causative role in cardiac fibrosis and hypertensive heart disease^[16]^. Thus, the strong human and mouse data from this new report, in combination with other recent students, will likely generate excitement about the potential role of PLA2G7 in cardiovascular aging.

## Figures and Tables

**Figure 1.**
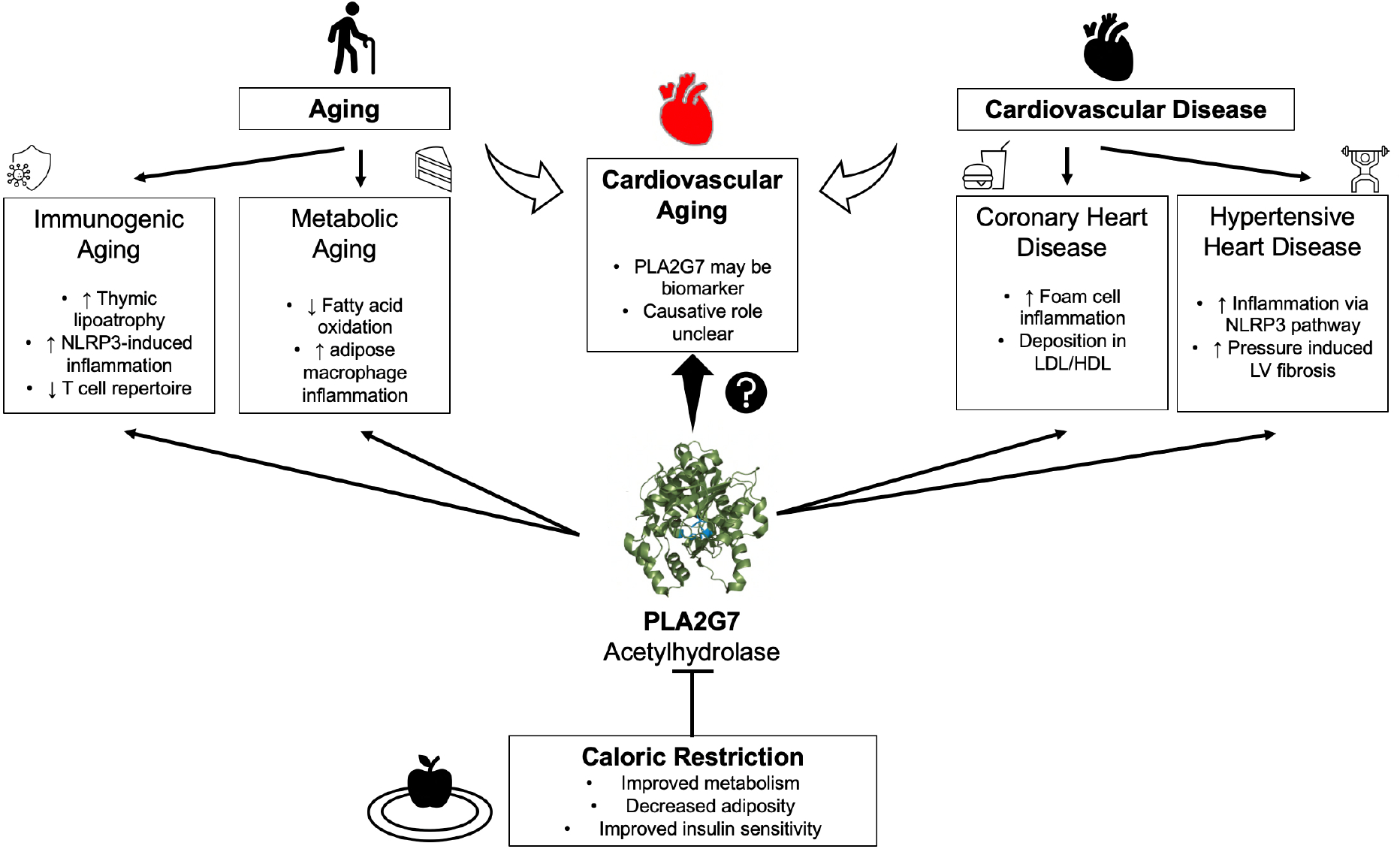
Prior work suggested a causative role of PLA2G7 in various cardiovascular diseases (right)^[4,16]^. Spadaro *et al.*^[3]^ demonstrated caloric restriction reduces PLA2G7 expression and improves immunogenic and metabolic aspects of aging (left). PLA2G7 age-related methylation has been suggested to be a biomarker of cardiovascular aging, though the mechanism is unclear, and PLA2G7 has not been shown to have a causative role in cardiovascular aging^[5]^.
